# Feasibility of Using Cranial Electrotherapy Stimulation for Pain in Persons with Parkinson's Disease

**DOI:** 10.4061/2010/569154

**Published:** 2010-05-05

**Authors:** Diana H. Rintala, Gabriel Tan, Pamela Willson, Mon S. Bryant, Eugene C. H. Lai

**Affiliations:** ^1^Research Service, Michael E. DeBakey Veterans Affairs Medical Center, Houston, TX 77030, USA; ^2^Department of Physical Medicine and Rehabilitation, Baylor College of Medicine, Houston, TX 77030, USA; ^3^Department of Anesthesiology, Baylor College of Medicine, Houston, TX 77030, USA; ^4^Parkinson's Disease Research, Education and Clinical Center, Michael E. DeBakey Veterans Affairs Medical Center, Houston, TX 77030, USA; ^5^Department of Neurology, Baylor College of Medicine, Houston, TX 77030, USA

## Abstract

*Objectives.* To assess the feasibility of treating musculoskeletal pain in the lower back and/or lower extremities in persons with Parkinson's disease (PD) with cranial electrotherapy stimulation (CES). *Design.* Randomized, controlled, double-blind trial. *Setting.* Veterans Affairs Medical Center, Community. *Participants*. Nineteen persons with PD and pain in the lower back and/or lower extremities. Thirteen provided daily pain rating data. *Intervention.* Of the thirteen participants who provided daily pain data, 6 were randomly provided with active CES devices and 7 with sham devices to use at home 40 minutes per day for six weeks. They recorded their pain ratings on a 0-to-10 scale immediately before and after each session. *Main Outcome Measure.* Average daily change in pain intensity. *Results.* Persons receiving active CES had, on average, a 1.14-point decrease in pain compared with a 0.23-point decrease for those receiving sham CES (Wilcoxon *Z* = −2.20, *P* = .028). *Conclusion.* Use of CES at home by persons with PD is feasible and may be somewhat helpful in decreasing pain. A larger study is needed to determine the characteristics of persons who may experience meaningful pain reduction with CES. Guidelines for future studies are provided.

## 1. Introduction

Reports of the prevalence of pain and other sensory symptoms in persons with PD have ranged from 34% to 85% [1–8]. Several authors have reported that pain may be the first presenting symptom of PD; however, it is only in retrospect that the relationship to PD becomes clear [3, 4, 6, 9–14]. Once antiparkinsonian medication is instituted, pain may fluctuate with “on” and “off” periods and with fluctuations in dystonia [7, 11]. In some cases, antiparkinsonian medication may initiate the pain or make it worse [11]. Pain may be chronic, become more prevalent, occur in a wider area of the body, and/or become more severe as PD progresses [7, 15, 16].

Various classification schemes have been suggested in reference to the etiology of sensory symptoms in PD. Based on category schemes previously developed by Goetz et al. [5] and Quinn et al. [11], Ford [7] proposed a modified 5-category scheme for classifying pain associated with PD: (a) musculoskeletal pain (pain due to rigidity, rheumatologic disease, or skeletal deformity), (b) neuropathic-radicular pain (pain due to root lesion, focal or peripheral neuropathy), (c) dystonic pain (related to abnormal tonicity and timing of dose of medication), (d) central pain (lesion or abnormality of function within the central nervous system and related to timing and dose of medication), and (e) akathisia (off period or drug-induced). Goetz et al. [5] found pain such as muscle cramps or tightness to be the most common (74% of patients with pain) followed by dystonias, usually in the feet (28%). Radicular, neuritic, joint, and generalized pains were less common (2% to 14%).

Occurrence and severity of pain may be related to other symptoms of PD such as tremor; rigidity; akinesia; dyskinesia, alterations in stance, gait, or mobility; postural deformities; radiculopathy; sciatica; myelopathy; and dystonia [7, 17]. Pain is also often associated with psychological conditions such as depression, anxiety, and sleep disorders [6, 7, 15, 17–20].

Recommended treatment for pain in patients with PD depends on the source of the problem [7]. If the pain is related to antiparkinsonian medication, the dose of the medication may need to be regulated upward or downward or a trial with a different antiparkinsonian medication may be indicated [7, 17, 21]. If the source of the pain is musculoskeletal in nature, physical therapy [7], exercise [7], oral appliances [22], nonsteroidal anti-inflammatory drugs [7], analgesics [7], botulism toxin [7, 23], and arthroscopic or orthopedic surgery [7, 24] may be indicated [17]. For other etiologies, tricyclic agents, gabapentin, opiates, and clozapine may be helpful [7, 9, 19, 22]. Honey et al. [25] found that PD-related pain was significantly reduced with unilateral pallidotomy. Loher et al. [26] found that unilateral pallidal deep brain stimulation relieved sensory symptoms best on the contralateral side but had a lesser effect on the ipsilateral side. Bilateral deep brain stimulation also was found to relieve sensory symptoms [26]. Concurrent psychological symptoms such as depression, anxiety, and sleeplessness may require treatment specific to the problem (e.g., antidepressants) in addition to other measures taken to relieve pain.

**Cranial Electrotherapy Stimulation (CES)**

CES is a noninvasive technique used to treat a variety of conditions involving the application of a small amount of electric current through the head via ear-clip electrodes. The analgesic action of subperception CES has been demonstrated in various antinociception models [27–29]. Extracellular recording techniques indicated that CES modifies noxious evoked responses in pain-processing regions of the brains of rats [30, 31]. In humans, the mechanism of action of CES is not fully understood; however, it has been shown to “normalize” neurotransmitter homeostasis [32], stimulate the hypothalamic-pituitary axis by increasing IGF-1 production [33], bring neurotransmitters in stressed participants back to normal levels of homeostasis [34], and increase *β*-endorphins in patients with chronic back pain [35].

A review of the use of CES in the management of chronic pain [36] concluded that CES has been found to be effective in decreasing spinal pain [37], headache pain [38–40], dental pain [41, 42], and pain-related muscle spasms [43–45], as well as controlling a variety of conditions that are often associated with pain—anxiety, depression, insomnia, and generalized stress [46]. CES has been found to have very few negative side effects. There have been reports of slight irritation at the electrode sites [39], burns at the electrode site [47], slight dizziness [43], headache [48], giddiness [48], and tooth pain [48]. In a recent study, Tan et al. found CES to be effective in relieving musculoskeletal and neuropathic pain in persons with spinal cord injury (SCI) [49]. Not only was there a significant difference in the decrease in pain from before to after daily sessions between those who received active CES versus those who received sham CES, but also there was a significant change in pain interference with several quality-of-life domains from pre- to postintervention.

To our knowledge there have been no studies published regarding the use of CES for pain management in the PD population. Therefore, we conducted a small initial study to determine (a) the feasibility of using CES at home to relieve chronic musculoskeletal pain in the lower back and/or lower extremities in the PD population, regardless of pain etiology, (b) whether a larger study is warranted, and (c) how to best design any future study of CES in the PD population. Musculoskeletal pain in the lower back and/or lower extremities was selected for the study because it is common among persons with PD [5], thus increasing the likelihood of recruiting a sufficient number of participants. Limiting the study to only lower body pain ensured a more homogeneous sample and lower body pain is more disabling because it affects mobility.

## 2. Methods

From July 2005 through June 2006 a convenience sample was recruited through the Parkinson's Disease Research, Education, and Clinical Center at the Michael E. DeBakey Veterans Affairs Medical Center (MEDVAMC), the newsletter of the Houston Area Parkinson Society, and word of mouth. This feasibility study was designed to include up to 20 participants. This sample size was selected based on the availability of resources to support a part-time study coordinator and other project costs. *Inclusion criteria* were (a) diagnosis of PD (diagnosis code of 332.0) confirmed by a movement disorder specialist, (b) at least one chronic musculoskeletal pain in the lower back and/or lower extremity (not necessarily PD-related pain) that began at least six months prior to study entry, (c) average pain intensity rated at the time of recruitment as at least 5 on a scale of 0 (no pain) to 10 (pain as bad as you can imagine), and (d) speak and understand English. *Exclusion criteria* were (a) current substance abuse problem, (b) currently under treatment for a serious psychological or psychiatric disorder that could affect ability to participate in the study, (c) moderate or severe cognitive impairment (score <21 on the Telephone Interview for Cognitive Status (TICS) [50]), (d) implanted electrical device (e.g., cardiac pacemaker, defibrillator, or deep brain stimulator), and (e) pregnancy. 

Participants signed an informed consent form approved by Baylor College of Medicine and the MEDVAMC. The participants completed questionnaires and were trained in the use of the CES device and Daily Pain Rating Sheet. They were randomized to receive either an active or a sham CES device for use at home during the study. 

**Figure 1:**
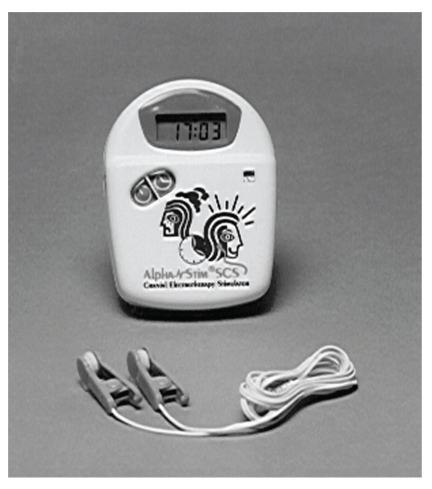
Cranial electrostimulation therapy (CES) device.

The CES equipment used was the Alpha-Stim SCS (Supplier: Electromedical Products International, Inc, 2201 Garrett Morris Parkway, Mineral Wells, TX 76067-9034 USA), which is a prescription medical technology that is Federal Drug Administration approved for the management of pain, anxiety, depression, and insomnia. The device is illustrated in Figure 1. Half of the devices provided active CES and the other half had no electric current flowing. Active devices provided subsensory stimulation of 100 microamperes. Active and sham devices were identical in appearance and were identified only with a serial number. Participants and the study coordinator were blinded to group assignment and the code sheet indicating which devices were active and which were sham was kept by another person who was not in contact with the participants. Participants in both the active and sham groups were taught how to use the device, provided with printed instruction sheets, and instructed to use the device 40 minutes daily for six weeks. They were provided with ear clip electrode pads, conducting solution with which to wet the pads, and 9-volt batteries.

A summary of the measures is provided in Table 1. Standardized scales included the TICS [50], the Hoehn and Yahr (HY) Staging of PD Scale [51], and the Unified Parkinson's Disease Rating Scale (UPDRS) [52]. The UPDRS motor scale was administered by a PD specialist. Demographic and PD data and information on pain and side effects were provided by the participants. The participants were instructed to provide daily pain ratings on a 0-to-10 scale immediately before and immediately after the end of each 40-minute CES session during the 42-day trial.

For each participant, average pre- and postsession pain ratings were calculated across the number of daily sessions completed. The difference between the average pre- and postsession ratings yielded an average mean change score. Differences in pain ratings between the active and sham groups were assessed with nonparametric Mann-Whitney *U* tests. Within groups, nonparametric Wilcoxon Signed Rank tests were performed to determine whether there was significant pre- to postsession change. The average percent of change from before to after sessions was calculated and Mann-Whitney *U* tests were performed to assess differences between groups.

## 3. Results

Nineteen participants were recruited and randomized to the active or sham condition. Nine (47.4%) participants had low back pain, two (10.5%) had lower extremity pain, seven (36.8%) had both low back and lower extremity pain, and the specific location of the “study pain” was missing for one (5.3%) participant. The mean number of days a month with pain was 26.3 ± 6.2 and the mean number of hours a day with pain was  7.6 ± 4.2. Characteristics of the sample can be seen in the column labeled “Overall” in Table 2. There were no significant demographic differences between the initial active (*n* = 9) and sham (*n* = 10) groups. There also was no significant difference between the groups in average pain intensity in the past week at the time of the screening telephone call or at the time of the initial visit to the MEDVAMC. 

Thirteen (68%) participants provided at least some daily pain data and the remainder of this report concerns data from these 13 participants. Half of the six who did not provide daily data had received active CES devices and half had received sham devices. Reasons for missing daily pain ratings were as follows: *Active—*never started using the device for an unknown reason (*n* = 1), frustrated, and concerned that he was not using it correctly, so stopped using it and did not return the pain rating sheet (*n* = 1), and based on weekly telephone contact data another participant used the device but did not return the daily pain rating sheet for an unknown reason (*n* = 1); and *Sham*—never started using the device because he moved (*n* = 1), did not understand how to complete the daily pain rating sheet, and only put checkmarks indicating that she used the device each day but did not rate pain intensity before and after each session (*n* = 1) and completed but then lost the rating sheet (*n* = 1). 

### 3.1. Daily Pain Ratings

Of the 13 recruits who returned at least some daily pain ratings, 6 were in the active group and 7 in the sham group. There was no significant difference in the demographic and PD data between those who did and did not return daily pain rating data or between the active and sham groups who provided daily data (Table 2). On average, the participants with data provided ratings for 31 (74%) of the 42 sessions planned for in the study protocol.

**Figure 2:**
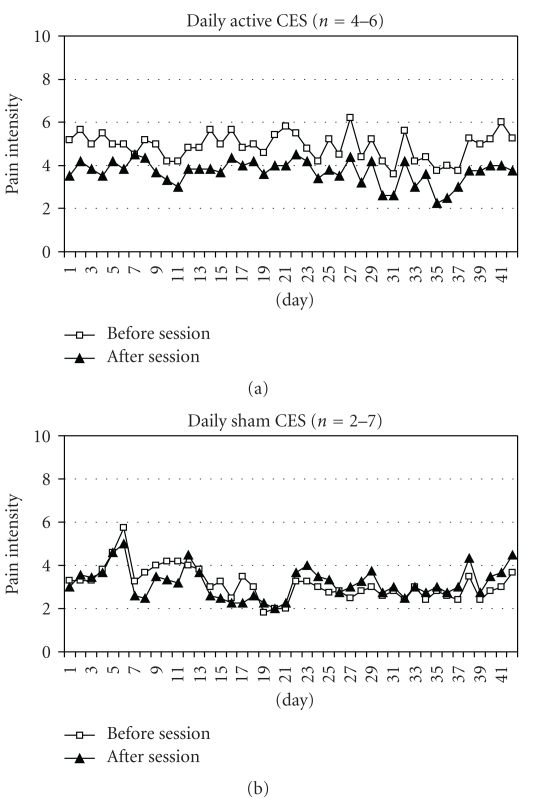
Average daily pain ratings before and after CES sessions for active and sham groups.

Displayed in Figure 2 are the average daily pain ratings for the active and sham groups. For the active group, the average daily rating was 4.89 ± 1.22 before and 3.75 ± 2.04 after the sessions yielding an average decrease of 1.14 ± 1.21 points (Wilcoxon *Z* = −2.20, *P* = .028). For the sham group, the average rating was 3.82 ± 1.76 before and 3.59 ± 1.75 after yielding an average decrease of 0.23 ± 0.33 (Wilcoxon *Z* = −1.36, *P* = .173). As can be seen in Figure 2, within each group, average daily change was quite consistent across time. The average difference between the groups in change scores (1.14 versus 0.23) was significant (Mann-Whitney *U* = 7.00, *P* = .045), indicating that pain reduction in the active group was greater than that in the sham group. 

Three (50%) of the six active CES participants with daily ratings had an average daily decrease in pain intensity of at least one point (3.41, 1.35, and 1.14). None of the sham group had a decrease of at least one point. The decreases in the active group represented 90, 29, and 21 percent, respectively, of their average before-session ratings. Decreases between 10 and 20 percent are considered minimally clinically important, decreases ≥30 percent are moderately clinically important, and decreases ≥50 percent are considered substantial [53]. Only one person in the sham group had a decrease of greater than 20 percent and he had a very low average before-session pain rating of 0.49. However, a Mann-Whitney *U* test revealed no significant difference between the groups on percentage change (*U* = 11.00, *P* = .153).

Side effects were minimal: *Active*—pulsing, tickling, or tingling sensations on ears (*n* = 3), tender ears (*n* = 1), and pins-and-needles sensation near the bladder (*n* = 1); and *Sham*—drowsiness (*n* = 1), warm ears (*n* = 1), and headache after one session (*n* = 1). Some participants had difficulty manipulating the ear clips and/or replacing the small electrode pads. No serious study-related adverse events occurred during this study.

## 4. Discussion

This small initial study examined the feasibility of using CES for chronic musculoskeletal pain in persons with PD. The participants were able to use the devices at home, sometimes with the help of a family member. Side-effects were mild and did not cause withdrawals. The results suggest that there may be some benefit from CES for some individuals. The consistency of the daily change scores across the 6-week trial suggests that the results were not due to a placebo effect. 

The primary limitations of the study are the small sample size and a large number of participants without daily pain ratings (32% of 19 recruits). The two participants who never started using the device were both males who rated their pain as a 5 on the 0-to-10 scale at intake (the minimum pain rating that met the criteria). However, many other participants who did use the device also had an initial pain rating of 5, suggesting that it may not have been less intense pain that influenced the nonuse of the device. Clearly, better training on the use of the daily pain rating sheet is needed to increase the likelihood of usable data. In addition, perhaps the daily rating sheet data should be obtained and recorded by project staff during weekly telephone contacts to avoid possible loss of data.

The small sample limits our ability to generalize the findings to other persons with PD who have musculoskeletal pain in the lower-body. Larger studies are needed to assess the effect of CES on pain in persons with PD to extend the findings of the present study. Furthermore, based on the data from the active group, it is clear that some participants had a better response to the treatment than others. Future studies should be large enough to assess differences between responders and nonresponders to identify any characteristics that may be predictive of successful pain management through the use of CES. 

In spite of meeting the criterion at the time of recruitment that pain intensity was rated as at least a 5 on a scale of 0 to 10, some participants had an average before-session pain intensity rating below 5 during the 6-week trial. The fact that the active group had approximately one point higher average before-session scores than the sham group, despite random assignment to groups, may have had an effect on the results although average change in pain intensity was not significantly related to the average before-session pain ratings (Spearman rho = − 0.15, *P* = .615). The percentage change was also not significantly related to the before-session pain rating (Spearman rho = 0.11, *P* = .720). In future studies, stratification based on initial pain ratings before randomization may improve the similarity of the two groups.

In spite of randomization, the active group was, on average, 10 years younger than the sham group at the time of diagnosis of PD and, time since diagnosis was 10 years longer for the active group at study entry, (Table 2). What effect these differences may have had on the results is unknown; however, neither change in pain intensity nor percentage of change in pain intensity was related to age at diagnosis (change in pain: Spearman rho = 0.28, *P* = .364; percentage change in pain: Spearman rho = 0.41, *P* = .162) or time since diagnosis (change in pain: Spearman rho = − 0.36, *P* = .231; percentage change in pain: Spearman rho = − 0.22, *P* = .471). In future studies, stratification on age at diagnosis and time since diagnosis may be wise in order to better equalize the groups.

Another apparent, but not significant difference between the groups was the UPDRS Mentation subscale (Table 2). However, since the possible total score on the subscale is 16 and the difference between the groups is only 1.5 points, this is not considered to be a meaningful clinical difference. Scores between 1 and 4 are considered to indicate mild conditions. Mentation was not significantly related to change in pain (Spearman rho = − .05, *P* = .878) or percentage change in pain (Spearman rho = − .33, *P* = .270). Furthermore, the groups had very similar scores on the TICS.

We did not do a manipulation check regarding how successful the blinding of the participants was. Asking the participants whether they believed they had an active or sham device would have been instructive on this point. Since the before to after session effect of active CES on pain remained relatively constant from week to week, it may not be necessary to have such a long trial in future studies. Persons initially assigned to sham CES should be given the opportunity to subsequently try open-label active CES. The results of the open-label phase could strengthen the conclusions, if CES is effective. 

Additional suggestions for future studies are as follows: (a) daily before-and-after session pain ratings should be included in any assessment of the effectiveness of CES to relieve pain in persons with PD and the duration of pain relief after the session, if any, should be assessed; (b) daily treatments should be 60 minutes long in studies that use the subsensory current level (i.e., 100 *μ*A) to better compensate for the low current; (c) trials are needed in which the strength of the electric current is set above subsensory levels to determine if there is a “dose” response; (d) improvements in the design of the ear clips to make it easier for persons with poor hand muscle control would be helpful for PD and other disability populations; (e) improvement in training participants to use the device at home would be helpful, such as provision of a DVD with instructions and demonstrations to be used at home.

## 5. Conclusion

In spite of the limitations, the present study met the goals of an initial study. Our findings suggest that using CES at home is feasible in the PD population without serious unintended effects and there may be a decrease in pain for some persons. A larger study is needed to extend our findings, and the study provided methodological guidance for future studies.

## Figures and Tables

**Table 1:** Summary of measures.

Measure	Purpose	Administration schedule
Demographic and PD Data	Describe participants and their PD (age, gender, race/ethnicity, marital status, level of education, age at which PD was diagnosed and time since PD diagnosis).	Preintervention
Telephone Interview for Cognitive Status	Exclude persons with moderate or severe cognitive impairment (scores <21)	Preintervention
Hoehn and Yahr Staging of Parkinson's Disease Scale	Classify stage of PD	Preintervention
Unified Parkinson's Disease Rating Scale	Measure mentation, behavior and mood, activities of daily living, motor function, and complications of therapy	Preintervention
Information on Pain	Describe pain location on body drawings; days per month and hours per day with pain. Rate average pain on a 0-to-10 scale scale with end points “no pain” and “pain as bad as you can imagine”	Preintervention
Daily Pain Rating Sheet	Measure current pain intensity immediately before and immediately after each daily session of active or sham CES (0-to-10 scale with end points “no pain” and “pain as bad as you can imagine”).	Daily during 6-week intervention (self-administered)

CES: Cranial Electrotherapy Stimulation; PD: Parkinson's disease.

**Table 2:** Characteristics of the sample.

Characteristic	Overall *N* = 19	Active with Daily Data *n* = 6	Sham with Daily Data *n* = 7
Demographic Information			
Age at study entry (mean ± SD)	72.4 ± 8.0	74.7 ± 7.8	74.4 ± 8.3
Gender—*n* (%)			
Male	15 (78.9)	4 (66.7)	6 (85.7)
Female	4 (21.1)	2 (33.3)	1 (14.3)
Race/Ethnicity—*n* (%)			
Caucasian	15 (78.9)	5 (83.3)	6 (85.7)
African American	1 (5.3)	0 (0.0)	0 (0.0)
Hispanic	3 (15.8)	1 (16.7)	1 (14.3)
Telephone Interview for Cognitive Status (TICS) (mean ± SD)	32.8 ± 3.1	32.5 ± 3.9	34.1 ± 2.0
Parkinson's Disease Information			
Age when diagnosed with PD (mean ± SD)	62.6 ± 11.1	59.5 ± 12.4	69.2 ± 9.4
Time since diagnosis at study entry (mean ± SD)	10.6 ± 8.6	15.2 ± 12.9	5.2 ± 2.4
Hoehn and Yahr stage of PD—*n* (%)			
1.5 Unilateral disease with axial involvement	1 (5.3)	0 (0.0)	1 (14.3)
2.0 Bilateral disease without impairment of balance	6 (31.6)	0 (0.0)	2 (28.6)
2.5 Mild bilateral disease with recovery on pull test	5 (26.3)	4 (66.7)	1 (14.3)
3.0 Mild to moderate bilateral disease, some postural instability, physically independent	6 (31.6)	1 (16.7)	3 (42.9)
4.0 Severe disability, still able to walk or stand unassisted	1 (5.3)	1(16.7)	0 (0.0)
Unified Parkinson's Disease Rating Scale (UPDRS) (mean ± SD)			
Mentation, Behavior, and Mood (maximum score = 16)	2.3 ± 1.9	1.8 ± 1.6	3.3 ± 1.9
Activities of Daily Living (maximum score = 52)	14.3 ± 5.1	14.7 ± 5.2	14.4 ± 5.0
Motor Exam (maximum score = 108)	28.5 ± 12.7	27.3 ± 11.2	26.4 ± 12.6
UPDRS Total Score (maximum score = 176)	47.5 ± 18.1	46.2 ± 15.5	46.1 ± 17.0
Complications of Therapy (maximum score = 23)	2.5 ± 2.0	2.3 ± 1.4	2.0 ± 1.3

PD: Parkinson's disease; SD: standard deviation.

## Abbreviations

ANOVA: Analysis of variance
CES: Cranial electrotherapy stimulation
HY: Hoehn and Yahr Staging of Parkinson's Disease Scale
MEDVAMC: Michael E. DeBakey Veterans Affairs Medical Center
PD: Parkinson's disease
TICS: Telephone Interview for Cognitive Status
UPDRS: Unified Parkinson's Disease Rating Scale.

## References

[1] Lewy FH (1923). *Die Lehre vom Tonus und der Bewegung: Zugleich Systematische Untersuchungen Zur Klinik, Physiologie, Pathologie und Pathogenese der Paralysis Agitans*.

[2] Sigwald J, Solignac J (1960). Manifestations douloureuses de la maladie de Parkinson et paresthesies provoquees par les neuroleptiques. *Semaine Des Hopitaux Paris*.

[3] Snider SR, Fahn S, Isgreen WP, Cote LJ (1976). Primary sensory symptoms in parkinsonism. *Neurology*.

[4] Koller WC (1984). Sensory symptoms in Parkinson’s disease. *Neurology*.

[5] Goetz CG, Tanner CM, Levy M, Wilson RS, Garron DC (1986). Pain in Parkinson’s disease. *Movement Disorders*.

[6] Marinković Z, Kostić V, Coviković-Sternić N, Marinković S (1990). Pain in patients with Parkinson disease. *Srpski Arhiv Za Celokupno Lekarstvo*.

[7] Ford B (1998). Pain in Parkinson’s disease. *Clinical Neuroscience*.

[8] Schenkman M, Cutson TM, Zhu CW, Whetten-Goldstein K (2002). A longitudinal evaluation of patients’ perceptions of Parkinson’s disease. *Gerontologist*.

[9] Sandyk R (1982). Back pain as an early symptom of Parkinson’s disease. *South African Medical Journal*.

[10] Schott GD (1985). Pain in Parkinson’s disease. *Pain*.

[11] Quinn NP, Koller WC, Lang AE, Marsden CD (1986). Painful Parkinson’s disease. *Lancet*.

[12] Riley D, Lang AE, Blair RD, Birnbaum A, Reid B (1989). Frozen shoulder and other shoulder disturbances in Parkinson’s disease. *Journal of Neurology, Neurosurgery, and Psychiatry*.

[13] Scott B, Borgman A, Engler H, Johnels B, Aquilonius SM (2000). Gender differences in Parkinson’s disease symptom profile. *Acta Neurologica Scandinavica*.

[14] Ito S, Asahina M, Asahina M, Oki T, Hattori T (2001). Severe chronic pain with allodynia in Parkinson’s disease: a case report. *Rinsho Shinkeigaku*.

[15] Shang AB, King SA (1991). Parkinson’s disease, depression, and chronic pain. *Hospital and Community Psychiatry*.

[16] Karlsen KH, Tandberg E, Årsland D, Larsen JP (2000). Health related quality of life in Parkinson’s disease: a prospective longitudinal study. *Journal of Neurology, Neurosurgery, and Psychiatry*.

[17] Waseem S, Gwinn-Hardy K (2001). Pain in Parkinson’s disease: common yet seldom recognized symptom is treatable. *Postgraduate Medicine*.

[18] Goetz CG, Wilson RS, Tanner CM, Garron DC (1986). Relationships among pain, depression, and sleep alterations in Parkinson’s disease. *Advances in Neurology*.

[19] Stein WM, Read S (1997). Chronic pain in the setting of Parkinson’s disease and depression. *Journal of Pain and Symptom Management*.

[20] Caap-Ahlgren M, Dehlin O (2001). Insomnia and depressive symptoms in patients with Parkinson’s disease: relationship to health-related quality of life. An interview study of patients living at home. *Archives of Gerontology and Geriatrics*.

[21] Sandyk R, Bamford CR, Iacono RP (1988). Pain and sensory symptoms in Parkinson’s disease. *International Journal of Neuroscience*.

[22] Minagi S, Matsunaga T, Shibata T, Sato T (1998). An appliance for management of TMJ pain as a complication of Parkinson’s disease. *Cranio*.

[23] Grazko MA, Polo KB, Jabbari B (1995). Botulinum toxin A for spasticity, muscle spasms, and rigidity. *Neurology*.

[24] Vince KG, Insall JN, Bannerman CE (1989). Total knee arthroplasty in the patient with Parkinson’s disease. *Journal of Bone and Joint Surgery. British*.

[25] Honey CR, Stoessl AJ, Tsui JK, Schulzer M, Calne DB (1999). Unilateral pallidotomy for reduction of parkinsonian pain. *Journal of Neurosurgery*.

[26] Loher TJ, Burgunder J-M, Weber S, Sommerhalder R, Krauss JK (2002). Effect of chronic pallidal deep brain stimulation on off period dystonia and sensory symptoms in advanced Parkinson’s disease. *Journal of Neurology, Neurosurgery, and Psychiatry*.

[27] Wilson OB, Hamilton RF, Warner RL (1989). The influence of electrical variables on analgesia produced by low current transcranial electrostimulation of rats. *Anesthesia and Analgesia*.

[28] Capel ID, Dorrell HM, Spencer EP (1990). The application of sub-perception electrical stimuli elicits a temporally distinct response from restraint stress: I. Antinociceptive characteristics. *Journal of Bioelectricity*.

[29] Gabis L, Shklar B, Baruch YK, Raz R, Gabis E, Geva D (2009). Pain reduction using transcranial electrostimulation: a double-blind “active placebo” controlled trial. *Journal of Rehabilitation Medicine*.

[30] Qiao J-T, Skolnick M, Dafny N (1988). Dorsal raphe and external electrical stimulation modulate noxious input to single neurons in nucleus parafascicularis thalami. *Brain Research Bulletin*.

[31] Dong W-Q, Wilson OB, Skolnick MH, Dafny N (1992). Hypothalamic, dorsal raphe and external electrical stimulation modulate noxious evoked responses of habenula neurons. *Neuroscience*.

[32] Pozos RS, Strack LF, White RK, Richardson AW, Reynolds DV, Sjoberg AE (1971). Electrosleep versus electroconvulsive therapy. *Neuroelectric Research*.

[33] Smith RB, Ryser CA The use of cranial electrotherapy stimulation (CES) in anti-aging medicine: great things we learn when research goes wrong.

[34] Gold MS, Pottash AL, Sternbach H, Barbaban J, Asunitto W Anti-withdrawal effect of alpha methyl dopa and cranial electrotherapy.

[35] Gabis L, Shklar B, Geva D (2003). Immediate influence of transcranial electrostimulation on pain and *β*-endorphin blood levels: an active placebo-controlled study. *American Journal of Physical Medicine and Rehabilitation*.

[36] Kirsch DL, Smith RB (2000). The use of cranial electrotherapy stimulation in the management of chronic pain: a review. *NeuroRehabilitation*.

[37] Kulkarni AD, Smith RB (2001). The use of microcurrent electrical therapy and cranial electrotherapy stimulation in pain control. *Clinical Practice of Alternative Medicine*.

[38] Brotman P (1989). Low-intensity transcranial electrostimulation improves the efficacy of thermal biofeedback and quieting reflex training in the treatment of classical migraine headache. *American Journal of Electromedicine*.

[39] Solomon S, Elkind A, Freitag F (1989). Safety and effectiveness of cranial electrotherapy in the treatment of tension headache. *Headache*.

[40] Romano TJ (1993). The usefulness of cranial electrotherapy in the treatment of headache in fibromyalgia patients. *American Journal of Pain Management*.

[41] Clark MS, Silverstone LM, Lindenmuth J (1987). An evaluation of the clinical analgesia/anesthesia efficacy on acute pain using the high frequency neural modulator in various dental settings. *Oral Surgery, Oral Medicine, Oral Pathology*.

[42] Hochman R (1988). Neurotransmitter modulator (TENS) for control of dental operative pain. *The Journal of the American Dental Association*.

[43] Forster S, Post BS, Benton JG (1963). Preliminary observations on electrosleep. *Archives of Physical Medicine and Rehabilitation*.

[44] Magora F, Beller A, Aladjemoff L, Magora A, Tannenbaum J (1965). Observations on electrically induced sleep in man. *British Journal of Anaesthesia*.

[45] Kirsch DL (2002). *The Science Behind Cranial Electrotherapy Stimulation*.

[46] Kirsch DL, Lerner FN, Weiner RS (1998). Electromedicine: the other side of physiology. *Innovations in Pain Management: A Practical Guide for Clinicians*.

[47] Marshall AG, Izard CE (1974). Cerebral electrotherapeutic treatment of depressions. *Journal of Consulting and Clinical Psychology*.

[48] Singh K (1967). Sleep inducing devices a clinical trial with a Russian machine. *International Journal of Neuropsychiatry*.

[49] Tan G, Rintala DH, Thornby JI, Yang J, Wade W, Vasilev C (2006). Using cranial electrotherapy stimulation to treat pain associated with spinal cord injury. *Journal of Rehabilitation Research and Development*.

[50] Brandt J, Spencer M, Folstein M (1988). The telephone interview for cognitive status. *Neuropsychiatry, Neuropsychology and Behavioral Neurology*.

[51] Hoehn MM, Yahr MD (1967). Parkinsonism: onset, progression and mortality. *Neurology*.

[52] Fahn S, Elton RL, Fahn S, Marsden CD, Calne DB, Goldstein M (1987). The unified Parkinson’s disease rating scale. *Recent Developments in Parkinson’s Disease*.

[53] Dworkin RH, Turk DC, Wyrwich KW (2008). Interpreting the clinical importance of treatment outcomes in chronic pain clinical trials: IMMPACT recommendations. *Journal of Pain*.

